# A Case of Synchronous Laparoscopic Surgery for Sigmoid Colon Cancer Inaccessible by Colonoscopy and Left Adrenal Pheochromocytoma

**DOI:** 10.1155/cris/7531559

**Published:** 2026-09-16

**Authors:** Yasuhito Tsubokawa, Takaharu Kato

**Affiliations:** ^1^ Department of Gastrointestinal Surgery, Higashi Saitama General Hospital Satte, Saitama, Japan

**Keywords:** laparoscopic surgery, pheochromocytoma, sigmoid colon cancer

## Abstract

We report the case of a 65‐year‐old man with sigmoid colon cancer who presented with left lower abdominal pain. Preoperative computed tomography (CT) revealed a tumor in the left adrenal gland. As the patient had no clinical symptoms suggestive of catecholamine excess, the lesion was initially suspected to be a left adrenal metastasis; however, endocrinological evaluation was performed to establish the differential diagnosis. According to the Endocrine Society Clinical Practice Guidelines, the patient was diagnosed with a left adrenal pheochromocytoma. To reduce overall surgical invasiveness and shorten the hospital stay, synchronous laparoscopic resection of the sigmoid colon cancer and the adrenal tumor was planned. The procedure was performed using five ports commonly utilized for left‐sided colectomy, with an additional epigastric port, resulting in a total of six ports. Postoperatively, the patient developed adhesive small bowel obstruction, which resolved with conservative management, and he was discharged on postoperative day 19. Simultaneous laparoscopic resection of sigmoid colon cancer and left adrenal pheochromocytoma is extremely rare. The present case provides valuable insight suggesting that safe one‐stage surgery may be feasible through meticulous perioperative planning and the use of a transperitoneal approach with a shared operative field. Further accumulation of similar cases is warranted to establish optimal treatment strategies.

## 1. Introduction

In patients with malignant tumors, the presence of an adrenal tumor is often interpreted as metastasis [1]. However, the possibility of a primary adrenal tumor should also be considered, making accurate differential diagnosis essential. Here, we report a case in which a preoperative diagnosis of pheochromocytoma was established and simultaneous laparoscopic resection of both pheochromocytoma and sigmoid colon cancer was safely performed. Accurate preoperative diagnosis, meticulous perioperative planning, and appropriate management of intraoperative hemodynamic changes enabled safe completion of the simultaneous laparoscopic procedure.

## 2. Case Presentation

**Figure 1:**
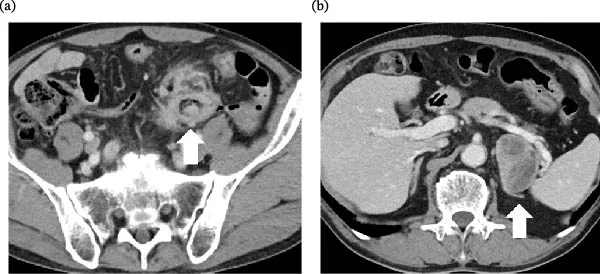
(a) Sigmoid colon cancer, and (b) A left adrenal tumor.

**Figure 2:**
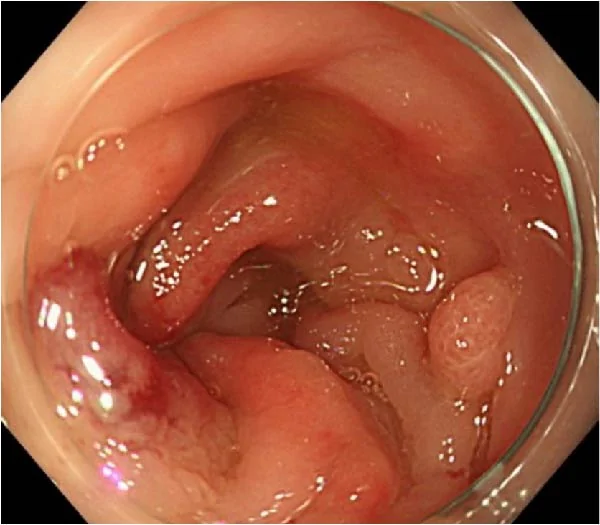
A circumferential sigmoid colon cancer with scope‐inaccessible stenosis.

**Figure 3:**
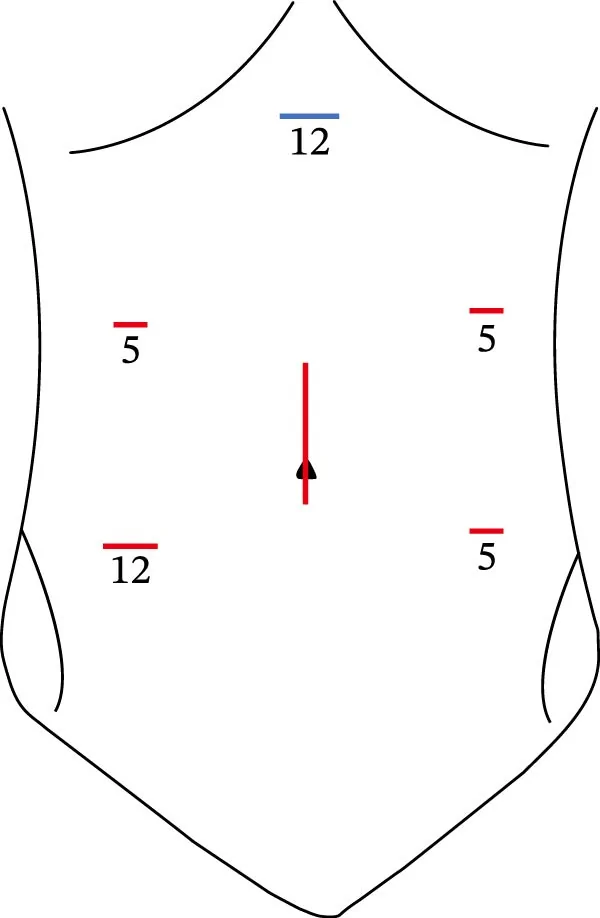
Port placement diagram.

The patient was a 65‐year‐old man who presented with left lower abdominal pain. Contrast‐enhanced computed tomography (CT) revealed findings suspicious for sigmoid colon cancer and a left adrenal tumor (Figure 1a,b). A colonoscopy was performed for further evaluation of the sigmoid colon lesion and revealed a circumferentially constricting tumor in the sigmoid colon that prevented passage of the endoscope (Figure 2). Endocrinological testing to evaluate the adrenal lesion revealed an elevated urinary metanephrine level of 1.65 mg/day, which was strongly suggestive of pheochromocytoma based on the Endocrine Society Clinical Practice Guidelines [2] (Table 1). To achieve a minimally invasive approach and avoid multiple hospitalizations, a single‐stage laparoscopic resection of the sigmoid colon cancer and the left adrenal tumor was planned. Preoperatively, the patient had already been receiving a calcium channel blocker prescribed at another hospital, and his blood pressure was well controlled. In addition, he had no apparent hypertensive episodes or clinical symptoms suggestive of pheochromocytoma. Therefore, no additional *α*‐adrenergic blockade or specific preoperative volume loading was performed in this case. The surgery was performed using six ports: five ports for left‐sided colectomy and an additional 12‐mm port placed in the epigastric region (Figure 3).

**Table 1:** The results of endocrinological testing.

Endocrinological test results					
			Hormonal levels in 24‐h urine collection		
TSH	1.48	μIU/mL	Urinary metanephrine	1.65	mg/day
Free T3	1.7	pg/mL	Urinary normetanephrine	0.94	mg/day
Free T4	1	pg/dL	Urinary adrenaline	48.2	μg/day
ACTH	12	pg/mL	Urinary noradrenaline	84	μg/day
Cortisol	13.1	μg/dL	Urinary dopamine	270.5	μg/day
Aldosterone	45	pg/mL	—	—	—
Plasma renin activity	<0.2	ng/mL/h	—	—	—
Dopamine	<10	pg/mL	—	—	—
Adrenaline	44	pg/mL	—	—	—
Noradrenaline	168	pg/mL	—	—	—

*Note:* Elevated urinary levels of metanephrine, normetanephrine, and adrenaline were observed in the 24‐h urine collection.

**Figure 4:**
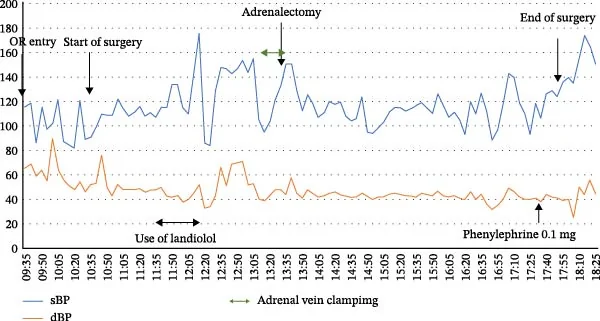
Perioperative blood pressure fluctuations.

**Figure 5:**
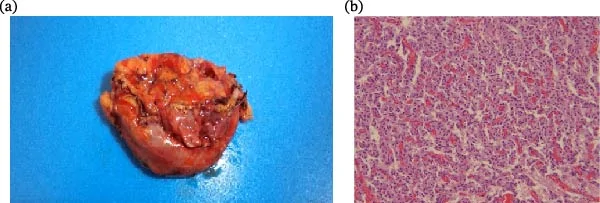
(a) Resected specimen of the adrenal tumor. (b) Microscopic findings (HE, medium magnification).

The patient was placed in the lithotomy position with the left shoulder elevated, and a left adrenalectomy was performed first. The lateral peritoneum of the splenic flexure was incised, followed by division of the lateral attachments of the spleen and incision of Gerota’s fascia to expose the anterior surface of the left adrenal gland. After division of the adhesions around the lower pole of the spleen, the plane between the pancreas and the transverse mesocolon was divided. During manipulation of the adrenal tumor, a transient increase in the heart rate was observed and was successfully controlled with the administration of landiolol. The adrenal vein was subsequently clamped for 1 min under continuous blood pressure monitoring, during which no significant hypotension was observed. After confirming hemodynamic stability, the adrenal vein was divided to minimize hemodynamic fluctuations caused by catecholamine release, followed by division of the adrenal arteries and subsequent resection of the left adrenal gland. A sigmoid colectomy was then performed, during which only minimal blood pressure fluctuations were observed, and the procedure was completed safely (Figure 4). Postoperative pathological examination confirmed that the adrenal tumor was a pheochromocytoma (Figure 5a,b). Although the patient developed adhesive small bowel obstruction postoperatively, it resolved with conservative treatment, and he was discharged on postoperative day 19. At 2 years and 5 months of follow‐up, the patient remains free of recurrence.

## 3. Discussion

Pheochromocytoma can cause significant intraoperative and postoperative blood pressure fluctuations as well as arrhythmias; therefore, meticulous preoperative preparation and careful perioperative management are required during surgical resection [3]. In particular, in cases complicated by malignant tumors, it is essential to evaluate on a case‐by‐case basis whether to resect the pheochromocytoma first or to proceed with simultaneous resection.

Pheochromocytoma is often not diagnosed during life and may remain clinically latent. Previous reports have shown that 41 of 54 cases (76%) were not diagnosed before death, and data from the Swedish cancer registry indicated that 41.9% of patients with pheochromocytoma (184 of 439 cases) were first diagnosed at autopsy [4, 5]. Because occult pheochromocytoma can cause sudden death during surgery, preoperative endocrinological evaluation is essential when an adrenal tumor is identified [2].

In patients with malignant tumors, adrenal tumors are sometimes presumed to represent metastases, and surgery may proceed without further investigation. However, the coexistence of pheochromocytoma and colorectal cancer has been reported, underscoring the importance of accurate differential diagnosis [6]. In our case, preoperative CT revealed sigmoid colon cancer and an adrenal tumor, raising the possibility of adrenal metastasis. However, endocrinological evaluation suggested pheochromocytoma, enabling careful preoperative management in close collaboration with anesthesiologists and allowing the surgery to be performed safely.

Sohawon et al. [6] suggested that staged surgery should be preferentially considered for patients with synchronous pheochromocytoma and colorectal neoplasia because of the potential for significant hemodynamic instability associated with pheochromocytoma. Furthermore, when synchronous surgery is undertaken, they recommended the posterior retroperitoneoscopic approach for adrenalectomy because it preserves the intraperitoneal dissection planes and may minimize postoperative intra‐abdominal adhesions [6].

In contrast, in the present case, synchronous surgery was selected to minimize surgical invasiveness after confirming that appropriate perioperative hemodynamic management could be achieved. With regard to the surgical approach, simultaneous sigmoid colectomy was planned in the present case, and mobilization of the splenic flexure was required to obtain sufficient bowel length for colorectal anastomosis. The transperitoneal approach allowed this mobilization to be utilized for both left adrenalectomy and sigmoid colectomy, enabling the two procedures to be performed sequentially through a shared operative field and common port placement. In addition, this approach avoided patient repositioning and the need for multiple additional flank ports required for the posterior retroperitoneoscopic approach, requiring only a single additional epigastric port (Figure 3). These findings suggest that, in patients undergoing simultaneous left‐sided colorectal resection and left adrenalectomy, the transperitoneal approach may represent a useful option because it offers both anatomical and procedural advantages by allowing the use of a shared operative field while minimizing patient repositioning and additional port placement. In addition, meticulous perioperative planning was considered essential to ensure the safety of the simultaneous procedure. Detailed preoperative discussions were held with the anesthesia team regarding the anticipated hemodynamic changes associated with tumor manipulation and adrenal vein control. Preparations for vasoactive agents, continuous arterial blood pressure monitoring, and prompt pharmacologic intervention were established before surgery. To minimize the impact of pheochromocytoma‐related hemodynamic instability on the subsequent colorectal procedure, left adrenalectomy was performed before sigmoid colectomy.

Traditionally, early adrenal vein control has been considered beneficial because it may reduce catecholamine release into the systemic circulation and suppress hemodynamic fluctuations associated with tumor manipulation. However, recent evidence suggests that adrenal vein control alone cannot completely prevent hemodynamic instability and that tumor manipulation itself is also an important contributing factor [7]. Accordingly, in this case, the tumor was manipulated carefully under continuous arterial blood pressure monitoring, and the adrenal vein was not immediately ligated and divided; instead, it was first clamped for 1 min to assess hemodynamic changes. If hypotension had occurred, the clamp could have been promptly released, while the anesthesia team had also prepared epinephrine and norepinephrine in advance for rapid pharmacologic intervention. After confirming the absence of significant hypotension, left adrenalectomy was completed, followed by sigmoid colectomy. This multidisciplinary strategy allowed the simultaneous laparoscopic procedure to be completed safely without major hemodynamic instability.

Although laparoscopic surgery is a minimally invasive approach, pheochromocytoma requires rapid responses to intraoperative hemodynamic changes, underscoring the importance of a comprehensive emergency response system involving the entire surgical team. Strategic port placement and meticulous preoperative planning, as demonstrated in this case, may contribute to improved safety in simultaneous surgical procedures.

However, simultaneous surgery for pheochromocytoma and other malignancies may result in prolonged operative time and more complex intraoperative hemodynamic management. In particular, blood pressure fluctuations during adrenal tumor manipulation require advanced monitoring and prompt pharmacologic intervention, necessitating close collaboration between the surgical and anesthesia teams. Therefore, careful case‐by‐case assessment is essential, with full consideration of these associated risks.

This case highlights that accurate preoperative diagnosis, meticulous perioperative planning, and a transperitoneal approach using a shared operative field may facilitate safe simultaneous laparoscopic left adrenalectomy and sigmoid colectomy in carefully selected patients. Because this report describes a single case, further studies are required to confirm the safety and generalizability of this strategy.

## 4. Conclusion

Adrenal masses detected in patients with colorectal cancer may be presumed to represent metastatic disease. However, because an unrecognized pheochromocytoma may cause serious perioperative complications, this diagnosis should be carefully excluded. Comprehensive endocrinological evaluation is therefore essential. In the present case, the preoperative diagnosis enabled meticulous perioperative planning and preparation for hemodynamic management, allowing synchronous laparoscopic surgery to be performed safely. However, because this report is based on a single case, these findings should be interpreted with caution, and further accumulation of similar cases is required to clarify the safety and usefulness of this surgical strategy.

## Funding

No funding was received for this manuscript.

## Consent

Written informed consent was obtained from the patient for publication of this case report and the accompanying images.

## Conflicts of Interest

The authors declare no conflicts of interest.

## Data Availability

The data that support the findings of this study are available upon request from the corresponding author. The data are not publicly available due to privacy or ethical restrictions.
